# Socioeconomic and health system determinants of zero-dose and under-immunization among children in Zambia: Evidence from the 2024 Demographic and Health Survey

**DOI:** 10.1371/journal.pgph.0006618

**Published:** 2026-10-30

**Authors:** Moses Mwale, Peter Jay Chipimo, Andrew Phiri, Patrick Sakubita, Penelope Masumbu, Bryson Musonda, Chipo Mwela, Kelvin Mwangilwa, Musole Chipoya, Priscilla Nkonde Gardner, Francis Dien Mwansa, Azmach Gebregiorgis, Muziya Chika, Kennedy Matanda, Freddie Masaninga, Jacob Sakala, Peter Clement Lugala

**Affiliations:** 1 World Health Organization Country Office for Zambia, Lusaka, Zambia; 2 Zambia National Public Health Institute, Lusaka, Zambia; 3 United Nations Children’s Fund (UNICEF) Zambia Country Office, Lusaka, Zambia; 4 Child Health and Nutrition Unit, Ministry of Health, Lusaka, Zambia; JIPMER PSM: Jawaharlal Institute of Post Graduate Medical Education and Research Department of Preventive and Social Medicine, INDIA

## Abstract

Despite progress in childhood immunization, Zambia retains pockets of zero-dose children (those who have not received the first dose of diphtheria-tetanus-pertussis-containing vaccine, DTP1) and under-immunised children (those missing one or more vaccines in the recommended schedule), sustaining vulnerability to vaccine-preventable diseases. Understanding the factors associated with missed vaccination is essential for achieving Immunization Agenda 2030 targets. We analysed cross-sectional data from 1,738 children aged 12–23 months identified through the 2024 Zambia Demographic and Health Survey, a nationally representative survey using a stratified two-stage cluster sampling design. Three outcomes were assessed: zero-dose status (no DTP1), under-immunization by the basic schedule (BCG, DTP1–3, OPV1–3, measles-1), and under-immunization by the national expanded schedule (basic plus PCV1–3 and rotavirus1–2). Survey-weighted multivariable regression identified associated factors. Nationally, 3.2% (95% CI: 2.1–4.3%) of children were zero-dose, 36.1% (95% CI: 33.0–39.2%) were under-immunised by the basic schedule, and 69.9% (95% CI: 67.0–72.8%) by the national schedule. Zero-dose prevalence ranged tenfold across provinces, from 0.6% in Lusaka to 6.9% in North Western. In adjusted models, children in the richest households had 93% lower odds of zero-dose than the poorest (aOR 0.07, 95% CI: 0.02–0.29). Facility delivery reduced zero-dose by 68% (aOR 0.32, 95% CI: 0.15–0.69). For basic-schedule under-immunization, secondary education (aOR 0.50, 95% CI: 0.30–0.82; prevalence ratio 0.68) and vaccination card possession (aOR 0.24, 95% CI: 0.14–0.40; prevalence ratio 0.53) were associated with lower odds. Wealth effects differed significantly by residence for all outcomes (p < 0.001). Zero-dose and under-immunization in Zambia are profoundly socioeconomically and geographically patterned. Reaching zero-dose children requires targeting the poorest households and those with home deliveries, particularly in North Western and Northern provinces. Improving vaccination card coverage may reduce broader under-immunization. Gaps in national schedule coverage are consistent with supply-side constraints requiring structural health system responses, though direct supply-side measurement was beyond the scope of this analysis.

## 1. Introduction

Immunisation is among the most cost-effective public health interventions, preventing millions of child deaths each year [1,2]. Recognising that its benefits remain unequally shared, the Immunization Agenda 2030 (IA2030) sets targets of 90% national and 80% district coverage for routine vaccines and a halving of the number of “zero-dose” children, those who have not received the first dose of a diphtheria–tetanus–pertussis-containing vaccine (DTP1), from 2019 levels [3]. The zero-dose child has become the central marker of the most marginalised populations. An estimated 14.3 million children were zero-dose in 2023, concentrated in South Asia and sub-Saharan Africa, and these children are multiply disadvantaged and at highest risk of vaccine-preventable disease [4–6].

Progress toward these targets remains uneven. Mean coverage of the first measles-containing dose (MCV1) across low- and middle-income countries (LMICs) was 79.2% in 2024, with modelling suggesting only 84.2% by 2030 [7]. This illustrates how far routine systems remain from equity targets even for a long-established antigen.

In Zambia, the Expanded Programme on Immunization schedules BCG at birth; three pentavalent (DTP-HepB-Hib), oral polio and pneumococcal conjugate (PCV) doses and two rotavirus doses in the first 14 weeks; and MCV1 at nine months. National DTP3 coverage reached approximately 87% by 2018 but masked substantial sub-national inequity, and the COVID-19 pandemic set back these gains [8,9]. Zambia has committed to IA2030 and to a national “Big Catch-Up” plan. The 2024 Zambia Demographic and Health Survey (ZDHS), capturing children born during the period of maximum pandemic disruption, provides recent nationally representative data with which to quantify immunisation gaps and their determinants. We aimed to (1) estimate the national and sub-national burden of zero-dose and under-immunised children, (2) identify socioeconomic and health-system factors independently associated with these outcomes, and (3) assess whether these determinants differ across the outcomes.

## 2. Methods

### Ethics statement

This secondary analysis used de-identified data from the 2024 Zambia Demographic and Health Survey (ZDHS), obtained from the DHS Program under its standard data use agreement and available to registered users on request. The dataset contains no identifiable participant information. A waiver of ethical approval for this secondary analysis was granted by the National Health Research Ethics Board of the National Health Research and Training Institute, Zambia. The original ZDHS 2024 protocol was approved by the institutional review boards of ICF, the National Health Research and Training Institute, and the National Health Research Authority, Zambia. During the original survey, verbal informed consent was obtained from all respondents before interview; data on the children included in this analysis were provided by their mothers, who gave verbal consent on their behalf.

### 2.1 Study design and data source

This study used cross-sectional data from the 2024 Zambia Demographic and Health Survey (ZDHS), a nationally representative household survey conducted by the Zambia Statistics Agency under the global DHS Programme. Zambia is a landlocked country in southern Africa with an estimated population of about 20 million and is administratively divided into ten provinces (Central, Copperbelt, Eastern, Luapula, Lusaka, Muchinga, Northern, North-Western, Southern and Western), which range from highly urbanised (Lusaka, Copperbelt) to predominantly rural. The ZDHS employs a stratified two-stage cluster sampling design. In stage one, primary sampling units (PSUs), enumeration areas, were selected with probability proportional to size from the 2022 national census sampling frame, stratified by province and urban or rural residence. In stage two, households were selected by systematic random sampling within each PSU. Data were collected across all ten provinces. Full survey methodology is described in the ZDHS 2024 final report [10].

### 2.2 Study population

The analytic population comprised children aged 12–23 months alive at the time of the survey. This age window is the standard DHS convention for immunization analysis, as it permits complete ascertainment of all vaccines scheduled during the first year of life (BCG at birth, DTP1 at 6 weeks, MCV1 at 9 months) while minimising recall bias. Covariates with missingness included antenatal care attendance (2.1%) and postnatal care (2.9%). This complete-case approach assumes data are missing at random conditional on observed covariates. We assessed this by comparing key characteristics between included and excluded children. Multiple imputation sensitivity analyses (Table C in S1 Appendix) yielded consistent results. The survey did not collect migration history for children, so migration was not considered as an exclusion criterion.

### 2.3 Outcome variables

Three binary immunization outcomes were constructed from vaccination histories using vaccination card data where available and maternal recall otherwise, consistent with standard DHS analytical conventions.

- *Zero-dose status*: no receipt of DTP1 (pentavalent vaccine first dose), per the WHO and UNICEF standard definition.
- *Under-immunization, basic schedule*: failure to receive one or more of BCG, DTP1–3, OPV1–3, and Measles-1, consistent with the standard WHO and DHS basic immunization indicator.
- *Under-immunization, national expanded schedule*: failure to receive one or more of the basic schedule plus PCV1–3 and Rotavirus1–2, reflecting Zambia’s full national EPI schedule at the time of the survey.

Vaccination was recorded as received if the card showed receipt or if the mother confirmed vaccination by recall. Missing or ‘don't know’ responses were coded as unvaccinated, consistent with the conservative DHS analytical convention.

### 2.4 Predictor variables

Predictor variables were selected through a two-stage process combining an a priori conceptual framework and data-driven screening. First, variables were identified based on the Tanahashi model of health service coverage [11] and systematic reviews of immunization determinants in sub-Saharan Africa [12,13], grouped into three domains:

- Socioeconomic and demographic characteristics: household wealth quintile [14], maternal education level, type of residence, province of residence, birth order, preceding birth interval, maternal age group, and child sex.
- Health service access variables: place of delivery (health facility versus home or other), distance to health facility perceived as a big problem, receipt of four or more antenatal care (ANC) visits, postnatal care (PNC) check for the child, vaccination card possession (card seen by interviewer), two or more tetanus injections during pregnancy, and household health insurance.
- Additional socio-behavioural predictors: religion (binary Christian versus non-Christian), marital status, maternal occupation, media exposure (any weekly radio, television, or newspaper), and financial barrier to treatment.

Second, a univariable screening was conducted for each candidate predictor against each outcome using survey-weighted logistic regression. Variables with p < 0.20 in univariable analysis, together with a priori confounders identified from the conceptual framework (household wealth quintile and place of residence), were entered into the multivariable models (Table A in S1 Appendix). This approach balances statistical efficiency with retention of variables considered theoretically important for immunization service access and equity, consistent with recommendations for observational studies in complex survey settings [15].

Because the primary objective was to identify determinants of immunization inequities rather than construct a purely predictive model, variables considered important within the Tanahashi framework were retained irrespective of statistical significance in univariable analyses where justified conceptually. Religion and household health insurance were not included in the zero-dose model because no zero-dose events occurred in the non-Christian or insured sub-groups; further detail is given in Table A in S1 Appendix.

### 2.5 Statistical analysis

The ZDHS 2024 recode files were obtained from the DHS Program in Stata format (.dta) and analysed in R version 4.6.1. A two-sided significance level of α = 0.05 was used throughout. All analyses accounted for the complex survey design of the ZDHS 2024. The child-level sampling weight was applied to produce nationally representative estimates. Primary sampling units were treated as clusters, and survey strata were defined using the DHS stratification variable. National and provincial prevalence estimates with 95% confidence intervals were calculated using survey-weighted methods.

Associations between predictors and immunization outcomes were examined using survey-weighted multivariable logistic regression. Adjusted odds ratios (aORs) with 95% confidence intervals were reported. For the rare outcome of zero-dose status (3.2% prevalence), odds ratios were retained as the primary effect measure because they closely approximate risk ratios when outcomes are uncommon. In contrast, for the high-prevalence outcomes of basic under-immunisation (36.1%) and national under-immunisation (69.9%), prevalence ratios (PRs) were estimated using survey-weighted Poisson regression with robust standard errors and were considered the primary measures for public health interpretation, as odds ratios increasingly overestimate relative associations when outcomes are common. Odds ratios for these outcomes are reported alongside PRs to facilitate comparison with previous literature.

Multicollinearity was assessed using generalised variance inflation factors (GVIF; Table B in S1 Appendix). Model calibration and influence diagnostics were evaluated using standard procedures.

Given the small number of zero-dose events (n = 59 in the complete-case sample), we adopted several approaches to minimise potential overfitting. First, we fitted a parsimonious model retaining only variables significant at p < 0.20 in univariable screening plus prespecified confounders. Second, we applied Firth penalised likelihood regression, which addresses separation and reduces small-sample bias in rare-event data. Third, multiple imputation was conducted to assess robustness to missing data (Table C in S1 Appendix). The consistency of findings across these approaches was used to assess the stability of model estimates.

Model fit was summarised with McFadden’s pseudo-R^2^ and a likelihood-ratio test against the null model. Because svyglm’s pseudo-likelihood has no valid log-likelihood, pseudo-R^2^ and the likelihood-ratio statistics were computed from the corresponding unweighted logistic models and are reported as approximations.

Effect modification by urban or rural residence was assessed through interaction terms and stratified analyses where statistically significant interactions were identified. Because significant wealth-by-residence interactions were observed, stratified analyses were conducted to characterise the nature and direction of these effects.

Mediation analyses were undertaken to assess whether health service access factors, particularly ANC attendance, explained socioeconomic differences in zero-dose status. These analyses were considered exploratory and intended to provide insight into potential pathways linking socioeconomic position and immunization outcomes; results are reported in Section 3.11.

Sensitivity analyses assessed robustness by addressing missing data through multiple imputation, applying penalised regression for rare outcomes, extending the eligible age range, excluding potential outlier provinces, and restricting analyses to card-verified vaccination records. Reporting followed STROBE guidelines for observational studies.

## 3. Results

### 3.1 Sample characteristics

A total of 1,738 children aged 12–23 months with complete outcome data were identified; 62 (3.6% unweighted; survey-weighted prevalence 3.2%, see §3.2) were zero-dose. After excluding 52 children (3.0%) with missing values on one or more covariates, 1,686 children (of whom 59 were zero-dose) remained for the multivariable models. Excluded and retained children were similar across measured background characteristics.

Two-thirds of the sample (67%) lived in rural areas, reflecting Zambia’s overall population distribution. About half of mothers (47.5%) had completed primary education, and half of households (50.7%) fell into the two poorest wealth categories. The vast majority of births occurred in health facilities (92.7%), and most mothers (84.5%) attended four or more antenatal care visits. Vaccination cards were available for review for 94.5% of children.

When we compared children who had received no DTP1 vaccine (zero-dose) with those who had, several differences emerged (Table 1). Zero-dose children were more likely to come from the poorest households, have mothers with less education, live in rural areas, be born outside health facilities, have mothers who attended fewer than four antenatal care visits, and lack vaccination cards. These differences were all statistically significant (p ≤ 0.027). Child sex, maternal age, and other characteristics were similar between groups.

**Table 1:** Baseline characteristics of children aged 12–23 months by immunisation status, Zambia DHS 2024 (N = 1,738).

Characteristic	Overall N = 1,738	Not zero-dose N = 1,676	Zero-dose N = 62	Fully imm. (basic) N = 1,112	Under-imm. (basic) N = 626	Fully imm. (national) N = 520	Under-imm. (national) N = 1,218
**Household wealth quintile**							
Poorest	475 (27%)	445 (27%)	30 (48%)	271 (24%)	204 (33%)	123 (24%)	352 (29%)
Poorer	406 (23%)	394 (24%)	12 (19%)	250 (22%)	156 (25%)	107 (21%)	299 (25%)
Middle	348 (20%)	332 (20%)	16 (26%)	209 (19%)	139 (22%)	99 (19%)	249 (20%)
Richer	292 (17%)	289 (17%)	3 (4.8%)	218 (20%)	74 (12%)	107 (21%)	185 (15%)
Richest	217 (12%)	216 (13%)	1 (1.6%)	164 (15%)	53 (8.5%)	84 (16%)	133 (11%)
**Maternal education**							
No education	134 (7.7%)	127 (7.6%)	7 (11%)	64 (5.8%)	70 (11%)	31 (6.0%)	103 (8.5%)
Primary	826 (48%)	783 (47%)	43 (69%)	481 (43%)	345 (55%)	220 (42%)	606 (50%)
Secondary	684 (39%)	673 (40%)	11 (18%)	494 (44%)	190 (30%)	226 (43%)	458 (38%)
Higher	94 (5.4%)	93 (5.5%)	1 (1.6%)	73 (6.6%)	21 (3.4%)	43 (8.3%)	51 (4.2%)
**Type of residence**							
Urban	581 (33%)	571 (34%)	10 (16%)	403 (36%)	178 (28%)	207 (40%)	374 (31%)
Rural	1,157 (67%)	1,105 (66%)	52 (84%)	709 (64%)	448 (72%)	313 (60%)	844 (69%)
**Province**							
Central	210 (12%)	202 (12%)	8 (13%)	138 (12%)	72 (12%)	72 (14%)	138 (11%)
Copperbelt	141 (8.1%)	138 (8.2%)	3 (4.8%)	97 (8.7%)	44 (7.0%)	51 (9.8%)	90 (7.4%)
Eastern	211 (12%)	202 (12%)	9 (15%)	131 (12%)	80 (13%)	61 (12%)	150 (12%)
Luapula	185 (11%)	181 (11%)	4 (6.5%)	114 (10%)	71 (11%)	40 (7.7%)	145 (12%)
Lusaka	178 (10%)	175 (10%)	3 (4.8%)	115 (10%)	63 (10%)	47 (9.0%)	131 (11%)
Muchinga	130 (7.5%)	128 (7.6%)	2 (3.2%)	84 (7.6%)	46 (7.3%)	35 (6.7%)	95 (7.8%)
Northern	201 (12%)	189 (11%)	12 (19%)	114 (10%)	87 (14%)	50 (9.6%)	151 (12%)
North-Western	160 (9.2%)	150 (8.9%)	10 (16%)	94 (8.5%)	66 (11%)	28 (5.4%)	132 (11%)
Southern	194 (11%)	189 (11%)	5 (8.1%)	138 (12%)	56 (8.9%)	74 (14%)	120 (9.9%)
Western	128 (7.4%)	122 (7.3%)	6 (9.7%)	87 (7.8%)	41 (6.5%)	62 (12%)	66 (5.4%)
**Place of delivery**							
Home / other	127 (7.3%)	112 (6.7%)	15 (24%)	70 (6.3%)	57 (9.1%)	25 (4.8%)	102 (8.4%)
Health facility	1,611 (93%)	1,564 (93%)	47 (76%)	1,042 (94%)	569 (91%)	495 (95%)	1,116 (92%)
**Antenatal care**							
< 4 ANC visits	262 (15%)	246 (15%)	16 (26%)	151 (14%)	111 (18%)	64 (13%)	198 (17%)
≥ 4 ANC visits	1,439 (85%)	1,394 (85%)	45 (74%)	938 (86%)	501 (82%)	447 (87%)	992 (83%)
Any postnatal check (yes)	501 (30%)	488 (30%)	13 (22%)	333 (31%)	168 (28%)	147 (29%)	354 (30%)
**Vaccination card**							
Not seen	95 (5.5%)	82 (4.9%)	13 (21%)	29 (2.6%)	66 (11%)	19 (3.7%)	76 (6.2%)
Card seen	1,643 (95%)	1,594 (95%)	49 (79%)	1,083 (97%)	560 (89%)	501 (96%)	1,142 (94%)
**Preceding birth interval**							
First-born	524 (30%)	503 (30%)	21 (34%)	349 (31%)	175 (28%)	158 (30%)	366 (30%)
< 24 months	112 (6.4%)	105 (6.3%)	7 (11%)	64 (5.8%)	48 (7.7%)	31 (6.0%)	81 (6.7%)
24–35 months	260 (15%)	251 (15%)	9 (15%)	149 (13%)	111 (18%)	66 (13%)	194 (16%)
36 + months	842 (48%)	817 (49%)	25 (40%)	550 (49%)	292 (47%)	265 (51%)	577 (47%)
**Maternal age group**							
< 20 years	240 (14%)	229 (14%)	11 (18%)	154 (14%)	86 (14%)	74 (14%)	166 (14%)
20–29 years	872 (50%)	843 (50%)	29 (47%)	562 (51%)	310 (50%)	264 (51%)	608 (50%)
30–39 years	502 (29%)	486 (29%)	16 (26%)	322 (29%)	180 (29%)	150 (29%)	352 (29%)
40 + years	124 (7.1%)	118 (7.0%)	6 (9.7%)	74 (6.7%)	50 (8.0%)	32 (6.2%)	92 (7.6%)
**Child’s sex**							
Male	847 (49%)	815 (49%)	32 (52%)	559 (50%)	288 (46%)	263 (51%)	584 (48%)
Female	891 (51%)	861 (51%)	30 (48%)	553 (50%)	338 (54%)	257 (49%)	634 (52%)

Values are n (column %). Not zero-dose vs zero-dose = received vs did not receive DTP1. Fully vs under-immunised (basic) = received all vs missing ≥1 of BCG, DTP1–3, OPV1–3 and MCV1. Fully vs under-immunised (national) = received all vs missing ≥1 of the basic schedule plus PCV1–3 and rotavirus1–2. “Any postnatal check” shows the number with any postnatal check. Descriptive p-values are omitted because covariate selection for the adjusted models followed the univariable screening in Table A in S1 Appendix. Maternal education (DHS v106): None = no formal schooling; Primary/Secondary = some or completed primary/secondary; Higher = any post-secondary education. Zero-dose events: 62 in this descriptive sample; 59 in the complete-case modelling sample (Table J in S1 Appendix).

### 3.2 National and provincial prevalence

Survey-weighted national estimates indicated that 3.2% of children aged 12–23 months had received no DTP1 vaccine (zero-dose). A much larger group, 36.1%, were missing at least one of the basic vaccines (BCG, DTP doses 1–3, polio doses 1–3, or measles vaccine). When the definition was expanded to include PCV and rotavirus vaccines (the national schedule), the proportion rose sharply: 69.9% of children were missing one or more nationally recommended vaccines.

Provincial variation was substantial across all three outcomes (Table 2; Fig 1). Zero-dose prevalence ranged from 0.6% in Lusaka Province to 6.9% in North Western Province, a more than tenfold difference. Basic schedule under-immunization ranged from 30.3% in Copperbelt to 43.9% in Northern. For the national schedule, the gap was also wide: from 52.9% in Western Province to 83.7% in North Western Province.

**Table 2:** Survey-weighted provincial prevalence of zero-dose and under-immunisation, children 12–23 months, Zambia DHS 2024.

Province	Zero-dose %	Zero-dose 95% CI	Basic UI %	Basic UI 95% CI	National UI %	National UI 95% CI
Central	3.7	1.0 to 6.5	34.9	27.9 to 41.9	65.4	59.0 to 71.9
Copperbelt	2.5	−0.2 to 5.3	30.3	22.3 to 38.4	62.2	53.5 to 71.0
Eastern	4.0	1.5 to 6.5	38.8	30.7 to 46.9	71.5	63.9 to 79.1
Luapula	2.1	0.1 to 4.1	38.7	32.1 to 45.4	78.8	71.8 to 85.8
Lusaka	0.6	−0.6 to 1.9	35.2	28.6 to 41.8	72.3	65.0 to 79.7
Muchinga	1.8	−0.7 to 4.3	35.7	27.4 to 44.0	73.0	65.4 to 80.6
Northern	5.8	2.5 to 9.1	43.9	34.8 to 53.0	76.0	69.7 to 82.3
North-Western	6.9	2.3 to 11.5	41.3	30.9 to 51.7	83.7	76.7 to 90.7
Southern	2.3	0.3 to 4.3	31.1	23.2 to 39.0	62.2	54.5 to 70.0
Western	4.5	1.2 to 7.8	32.2	21.9 to 42.6	52.9	42.4 to 63.4

Survey-weighted prevalence with 95% Wald confidence intervals accounting for the ZDHS 2024 complex design (sampling weights, strata, primary sampling units). Negative lower bounds arise from the Wald method in sparse strata. UI = under-immunised. Zero-dose = no DTP1; basic UI = missing ≥1 of BCG, DTP1–3, OPV1–3, MCV1; national UI = missing ≥1 of the basic schedule plus PCV1–3 and rotavirus1–2.

**Fig 1:**
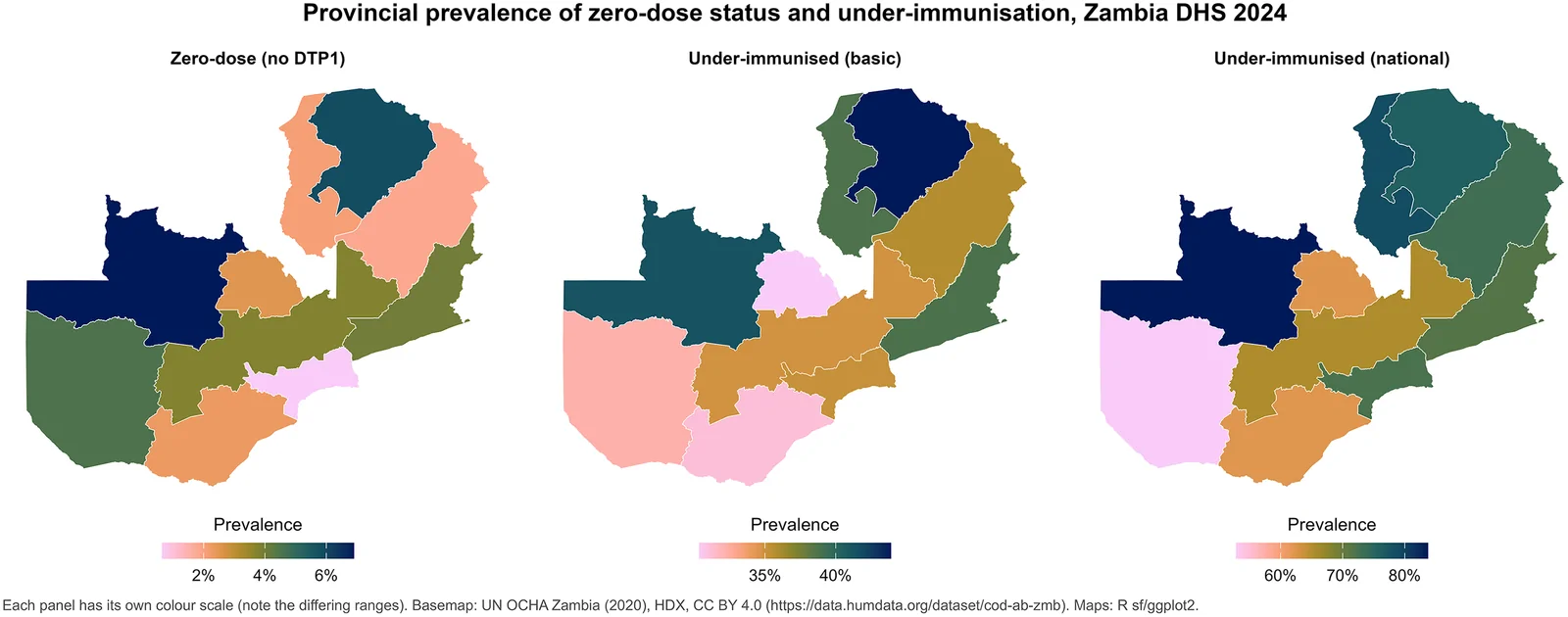
Provincial prevalence of zero-dose status and under-immunisation among children aged 12–23 months, Zambia DHS 2024. Base map shapefile: UN OCHA Zambia (2020), Humanitarian Data Exchange, CC BY 4.0 (https://data.humdata.org/dataset/cod-ab-zmb).

### 3.3 Prevalence ratio analysis for high-prevalence outcomes

Because under-immunisation according to the basic schedule (36.1%) and national schedule (69.9%) were common outcomes, prevalence ratios were estimated in addition to odds ratios. As expected, prevalence ratios were attenuated relative to odds ratios but demonstrated similar patterns of association.

For basic-schedule under-immunisation, maternal secondary education was associated with a 32% lower prevalence of under-immunisation (PR = 0.68, 95% CI: 0.52–0.89), while possession of a vaccination card was associated with a 47% lower prevalence (PR = 0.53, 95% CI: 0.44–0.63). Children from richer households also had lower prevalence of under-immunisation (PR = 0.71, 95% CI: 0.52–0.99).

For national-schedule under-immunisation, maternal higher education remained independently associated with lower prevalence (PR = 0.76, 95% CI: 0.59–0.98), whereas household wealth was not significantly associated after adjustment. Full comparisons between odds ratios and prevalence ratios are presented in Table D in S1 Appendix.

### 3.4 Factors associated with zero-dose status

Adjusted odds ratios for all three outcomes are presented in Table 3 and summarised in Fig 2. After accounting for all other factors, household wealth emerged as the strongest predictor of zero-dose status, with a clear dose-response pattern. Compared to children in the poorest households:

**Table 3:** Multivariable adjusted odds ratios for zero-dose status and under-immunisation (basic and national schedules), children 12–23 months, Zambia DHS 2024 (N = 1,686).

Characteristic	ZD aOR	ZD 95% CI	ZD p	Basic aOR	Basic 95% CI	Basic p	Nat. aOR	Nat. 95% CI	Nat. p
**Household wealth quintile**									
Poorest	1.00	(ref)		1.00	(ref)		1.00	(ref)	
Poorer	0.47	0.23–0.94	0.034*	0.95	0.68–1.33	0.768	1.08	0.76–1.55	0.668
Middle	0.66	0.30–1.43	0.292	0.90	0.63–1.28	0.546	0.92	0.63–1.37	0.693
Richer	0.22	0.05–0.87	0.031*	0.59	0.36–0.98	0.041*	0.86	0.52–1.44	0.568
Richest	0.07	0.02–0.29	<0.001***	0.56	0.31–1.01	0.055	0.85	0.45–1.57	0.596
**Maternal education**									
No education	1.00	(ref)		1.00	(ref)		1.00	(ref)	
Primary	1.25	0.54–2.89	0.609	0.70	0.45–1.09	0.115	0.86	0.52–1.42	0.554
Secondary	0.69	0.24–1.98	0.493	0.50	0.30–0.82	0.007**	0.73	0.42–1.29	0.283
Higher	2.40	0.42–13.75	0.323	0.46	0.22–0.95	0.036*	0.44	0.21–0.93	0.031*
**Type of residence**									
Urban	1.00	(ref)		1.00	(ref)		1.00	(ref)	
Rural	0.93	0.40–2.15	0.866	0.91	0.64–1.31	0.622	1.17	0.80–1.72	0.410
**Birth order**									
1st	1.00	(ref)		1.00	(ref)		1.00	(ref)	
2–3	0.56	0.24–1.31	0.181	1.00	0.72–1.39	0.999	0.73	0.51–1.05	0.087
4–5	0.53	0.19–1.48	0.222	0.98	0.63–1.54	0.936	0.88	0.56–1.39	0.589
6+	0.82	0.20–3.31	0.775	1.17	0.64–2.12	0.610	0.99	0.52–1.88	0.976
**Maternal age group**									
< 20 years	1.00	(ref)		1.00	(ref)		1.00	(ref)	
20–29 years	1.16	0.48–2.83	0.743	1.07	0.70–1.64	0.743	1.22	0.80–1.86	0.349
30–39 years	1.35	0.37–4.94	0.652	1.08	0.63–1.85	0.790	1.27	0.71–2.29	0.417
40 + years	1.12	0.22–5.77	0.896	0.95	0.47–1.90	0.881	1.28	0.60–2.70	0.522
**Child’s sex**									
Male	1.00	(ref)		1.00	(ref)		1.00	(ref)	
Female	0.92	0.55–1.54	0.746	1.28	1.03–1.60	0.025*	1.13	0.89–1.43	0.316
**Health service access**									
Facility delivery	0.32	0.15–0.69	0.004**	0.95	0.60–1.52	0.837	0.79	0.50–1.26	0.321
Distance a big problem	0.72	0.39–1.30	0.272	1.01	0.80–1.28	0.911	0.96	0.75–1.24	0.758
≥ 4 ANC visits	0.69	0.36–1.30	0.245	0.78	0.58–1.05	0.099	0.77	0.55–1.08	0.130
Any postnatal check	0.56	0.26–1.18	0.126	0.94	0.72–1.23	0.648	1.09	0.83–1.44	0.529
Vaccination card seen	—	—	—	0.24	0.14–0.40	<0.001***	0.65	0.37–1.13	0.124
Household size	1.01	0.93–1.11	0.744	1.00	0.95–1.05	0.850	0.96	0.91–1.00	0.072
**Other covariates**									
Non-Christian (vs Christian)	—	—	—	1.14	0.29–4.47	0.846	0.32	0.09–1.14	0.079
Married/cohabiting	—	—	—	1.13	0.76–1.69	0.532	1.25	0.83–1.87	0.280
Previously married	—	—	—	1.40	0.87–2.26	0.169	1.01	0.60–1.69	0.972
White collar / services	—	—	—	1.00	0.74–1.34	0.988	1.25	0.91–1.70	0.166
Agricultural	—	—	—	0.81	0.62–1.07	0.138	1.08	0.77–1.53	0.652
Manual labour	—	—	—	0.79	0.48–1.29	0.349	1.10	0.64–1.89	0.734
Financial barrier	—	—	—	0.97	0.53–1.77	0.928	1.42	0.76–2.64	0.272
≥ 2 tetanus injections	—	—	—	0.98	0.78–1.24	0.873	0.91	0.70–1.19	0.493

Adjusted odds ratios (aOR) with 95% confidence intervals from survey-weighted multivariable logistic regression (ZDHS 2024 complex design). Each outcome column is a single model. ZD = zero-dose; Nat. = national. Reference categories = 1.00 (ref). “—” = not included in that model: the marital-status, occupation, financial-barrier, vaccination-card and tetanus terms were not entered in the zero-dose model, and religion and health insurance were additionally excluded from the zero-dose model because of quasi-complete separation (no zero-dose events in one category). * p < 0.05; ** p < 0.01; *** p < 0.001. Marital status ref = never married; occupation ref = not working. For the common outcomes (basic 36%, national 70%) prevalence ratios are the primary effect measure and are given in Table D in S1 Appendix.

**Fig 2:**
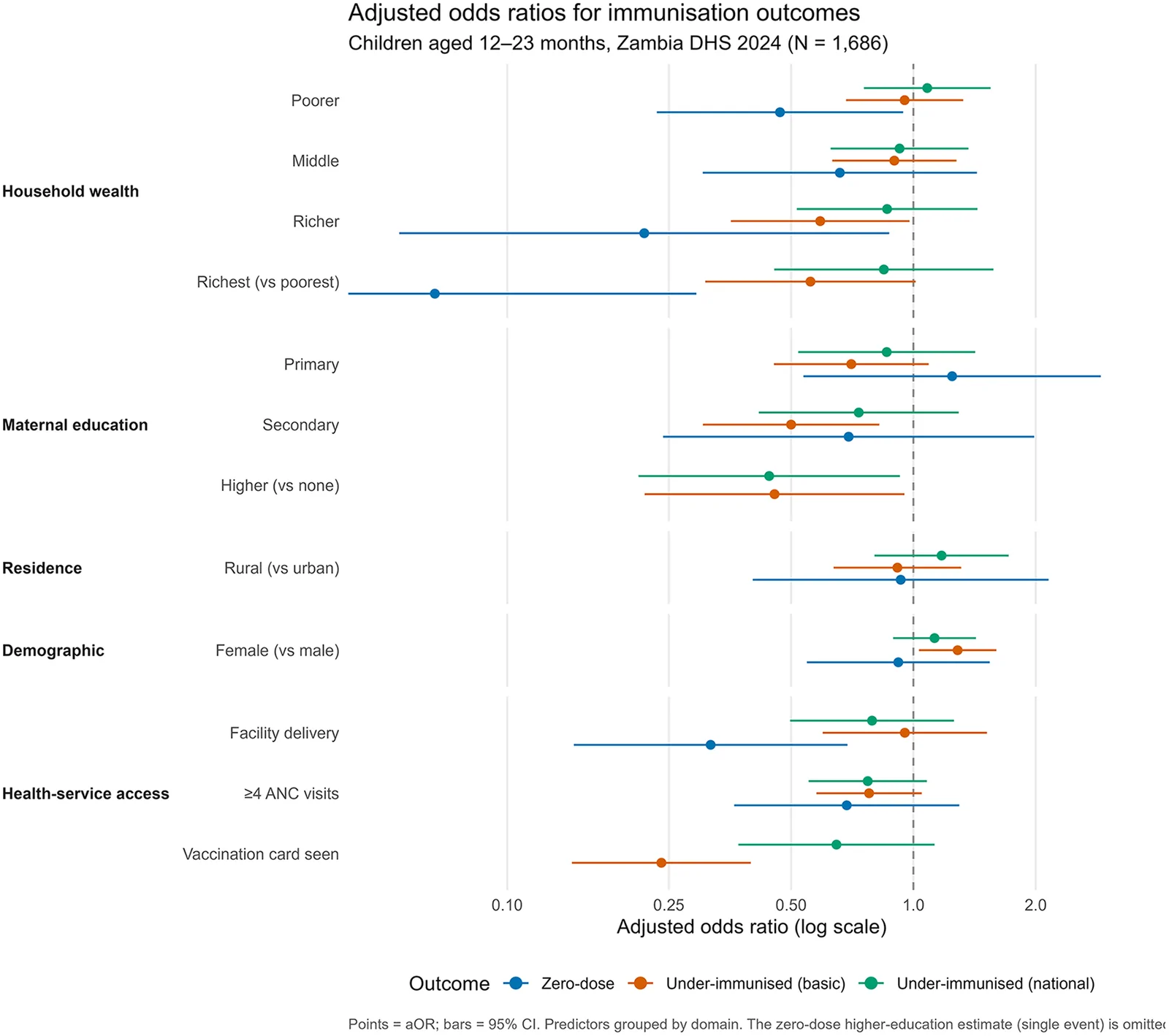
Forest plot of adjusted odds ratios for immunisation outcomes, children aged 12–23 months, Zambia DHS 2024 (N = 1,686). Adjusted odds ratios (points) and 95% confidence intervals (bars) on a log scale for selected socioeconomic, demographic and health-service predictors, shown separately for the three outcomes.

- Children in poorer households had 53% lower odds of being zero-dose (aOR 0.47, 95% CI: 0.23–0.94, p = 0.03)
- Those in richer households had 78% lower odds (aOR 0.22, 95% CI: 0.05–0.87, p = 0.03)
- Children in the richest households had 93% lower odds (aOR 0.07, 95% CI: 0.02–0.29, p < 0.001)

Once wealth and access to services were accounted for, maternal education no longer independently predicted zero-dose status. Similarly, rural residence became non-significant (aOR 0.93, 95% CI: 0.40–2.15, p = 0.87), suggesting that the rural disadvantage observed in unadjusted comparisons operates primarily through poverty and limited service access.

Place of delivery was independently associated with zero-dose status. Children born in health facilities had 68% lower odds of being zero-dose compared with those born at home or elsewhere (aOR 0.32, 95% CI: 0.15–0.69, p = 0.004). Antenatal care attendance, postnatal care, and perceived distance to health facilities did not independently predict zero-dose status once other factors were considered.

Results were robust to all sensitivity analyses. Firth penalised likelihood regression preserved the wealth gradient and facility delivery associations (Table L in S1 Appendix). Multiple imputation results were consistent with complete-case estimates (Table C in S1 Appendix). The Hosmer-Lemeshow test did not indicate poor model fit (χ² = 7.66, df = 8, p = 0.47; Table F in S1 Appendix).

### 3.5 Factors associated with missing basic vaccines

For the basic vaccine schedule, a different pattern emerged. Maternal education became an important factor. Compared to children whose mothers had no formal education:

- Those whose mothers had secondary education had 50% lower odds of missing basic vaccines (aOR 0.50, 95% CI: 0.30–0.82, p = 0.007; PR = 0.68)
- Those with mothers who had higher education had 54% lower odds (aOR 0.46, 95% CI: 0.22–0.95, p = 0.04; PR = 0.64)

Children in richer households also had lower odds of missing basic vaccines (aOR 0.59, 95% CI: 0.36–0.98, p = 0.04; PR = 0.71), while those in the richest households showed a similar but borderline effect (aOR 0.56, 95% CI: 0.31–1.01, p = 0.055; PR = 0.69).

The single strongest predictor was vaccination card possession. Children whose card was seen by the survey team had 76% lower odds of missing basic vaccines compared to those without a visible card (aOR 0.24, 95% CI: 0.14–0.40, p < 0.001; PR = 0.53). As both wealth and vaccination card possession are included simultaneously in the model, this association reflects the independent contribution of card possession net of socioeconomic status. On the absolute scale, the model-predicted probability of missing a basic vaccine was roughly 32–34 percentage points lower among children whose card was seen, a gap of similar magnitude across all wealth quintiles.

Girls had slightly higher odds of missing basic vaccines than boys (aOR 1.28, 95% CI: 1.03–1.60, p = 0.03), though this difference did not appear in the national schedule analysis.

### 3.6 Factors associated with missing national schedule vaccines

For the expanded national schedule (including PCV and rotavirus), the model had substantially less discriminatory ability, not unexpected given that 69.9% of children were missing at least one of these vaccines. After accounting for all factors, only maternal higher education remained associated with lower odds of national schedule under-immunization (aOR 0.45, 95% CI: 0.21–0.97, p = 0.05; PR = 0.76). No wealth category, place of residence, delivery location, or other access factor reached statistical significance.

### 3.7 Effect modification by residence

Statistical tests confirmed that the relationship between wealth and vaccination status differed significantly between urban and rural areas for all three outcomes (Table 4). For zero-dose children, the interaction was particularly strong (F = 179.2, p < 0.001). In contrast, the effect of facility delivery was consistent across urban and rural settings for zero-dose and basic schedule outcomes.

**Table 4:** Wald tests for effect modification by residence, Zambia DHS 2024.

Outcome	Interaction term	F statistic	p-value	Interpretation
Zero-dose	Wealth × residence	179.2	< 0.001	Significant
Zero-dose	Facility delivery × residence	0.08	0.783	Not significant
Under-immunised (basic)	Wealth × residence	36.0	< 0.001	Significant
Under-immunised (basic)	Facility delivery × residence	0.66	0.417	Not significant
Under-immunised (national)	Wealth × residence	42.3	< 0.001	Significant
Under-immunised (national)	Facility delivery × residence	3.24	0.072	Borderline

F statistics from survey-weighted Wald tests of the interaction term (regTermTest). The very large zero-dose Wealth × residence F is inflated by sparse urban zero-dose cells (only 10 urban zero-dose children) and should be read as evidence of the presence, not the magnitude, of effect modification.

When examining basic vaccine completion separately by residence, vaccination card possession was the dominant predictor in both settings, though somewhat stronger in urban areas. Maternal secondary education was strongly associated with completion in rural areas but not in urban areas.

The very large odds ratios for wealth in the urban zero-dose subgroup analysis should be interpreted with caution. With only 10 zero-dose children in urban areas, these estimates reflect the extreme rarity of the outcome in that subgroup rather than precise effect sizes.

### 3.8 Model stability and overfitting assessment

The complete-case zero-dose model included 59 events and 20 parameters, corresponding to an events-per-variable (EPV) ratio of 2.95. Although this is below the conventional recommendation of 10 events per parameter, findings were highly consistent across alternative modelling approaches.

A parsimonious model restricted to variables identified through univariable screening and a priori confounders yielded similar effect estimates. Facility delivery remained associated with substantially lower odds of zero-dose status (aOR=0.36, 95% CI: 0.18–0.74), while children in the richest households continued to demonstrate markedly lower odds than those in the poorest households (aOR=0.10, 95% CI: 0.02–0.42).

Firth penalised regression produced directionally consistent estimates and preserved the observed wealth gradient and facility-delivery effects. Multiple-imputation analyses also generated similar results. Collectively, these findings suggest that the principal conclusions are robust despite the limited number of zero-dose events.

### 3.9 Which specific vaccines are most often missed?

To identify which vaccines drive the 69.9% national schedule under-immunisation, we examined coverage for each antigen individually (Table 5; Fig 3).

**Table 5:** Survey-weighted antigen-level coverage and percentage missing, children 12–23 months, Zambia DHS 2024.

Vaccine (dose)	Coverage % (95% CI)	% Missing
BCG	94.7 (93.5–95.9)	5.3
Pentavalent (DTP-HepB-Hib) 1	96.8 (95.9–97.6)	3.2
Pentavalent 2	97.0 (96.1–97.8)	3.0
Pentavalent 3	94.7 (93.4–95.9)	5.3
OPV 1	93.5 (92.2–94.8)	6.5
OPV 2	90.4 (88.7–92.0)	9.6
OPV 3	78.3 (75.9–80.7)	21.7
Measles (MCV1)	87.2 (85.4–89.1)	12.8
PCV 1	94.6 (93.3–95.8)	5.4
PCV 2	92.1 (90.6–93.5)	7.9
PCV 3	86.6 (84.7–88.4)	13.4
Rotavirus 1	67.9 (65.2–70.5)	32.1
Rotavirus 2	53.2 (50.3–56.0)	46.8

Survey-weighted coverage (proportion receiving the dose) with 95% confidence intervals and the complementary percentage missing. Pentavalent = DTP–HepB–Hib; OPV = oral polio vaccine; MCV1 = first measles-containing dose; PCV = pneumococcal conjugate vaccine.

**Fig 3:**
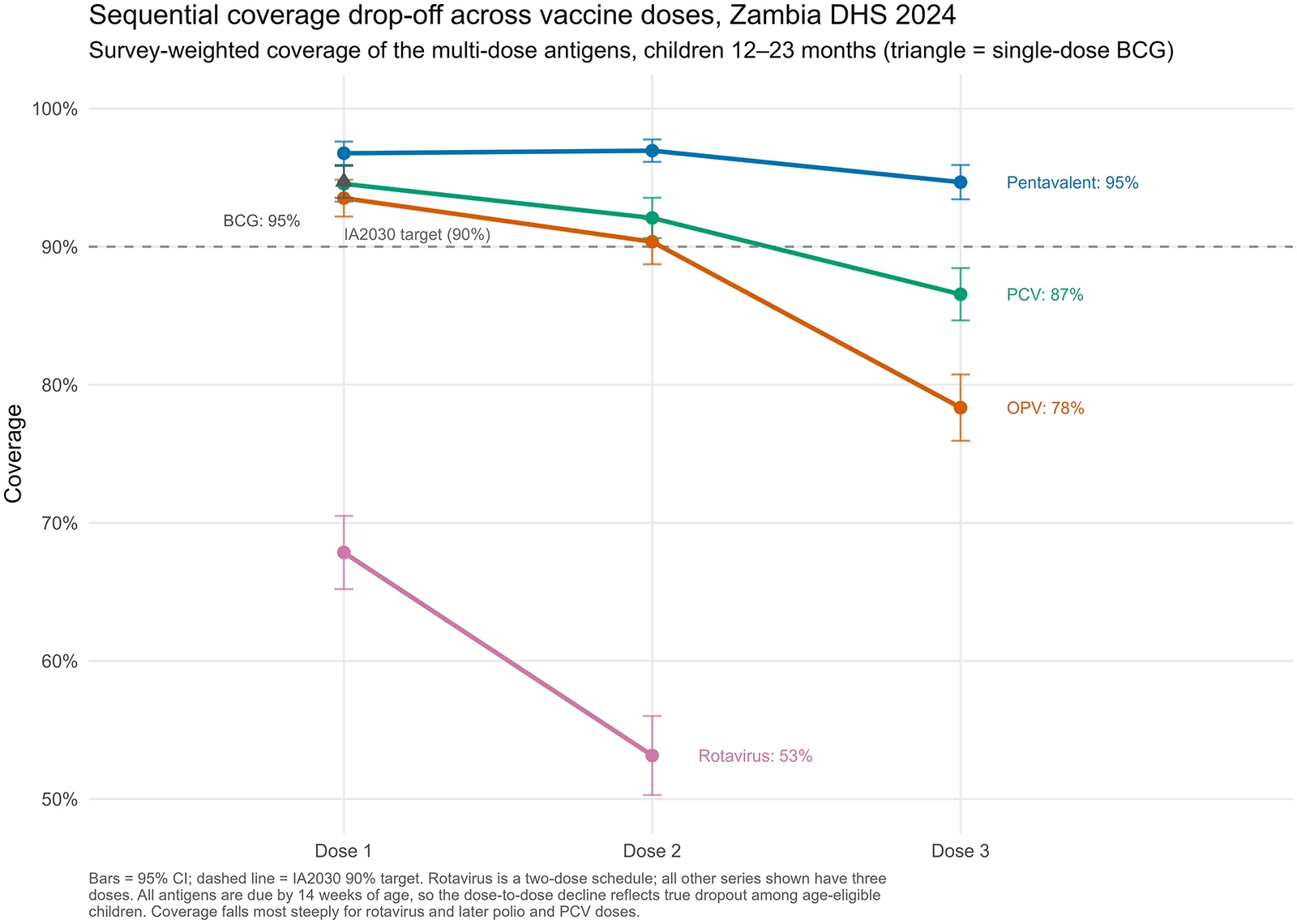
Sequential coverage drop-off across vaccine doses, Zambia DHS 2024. Survey-weighted coverage (%) with 95% confidence intervals for the multi-dose antigens among children aged 12–23 months; the triangle marks single-dose BCG.

Among the children who were under-immunised on the national schedule, the antigens they most often lacked were rotavirus dose 2 (missing in 67.7% of under-immunised children), rotavirus dose 1 (46.6%), polio dose 3 (30.4%) and PCV dose 3 (19.1%). Expressed as a share of all children (Table 5), the corresponding coverage gaps were 46.8%, 32.1%, 21.7% and 13.4%. Coverage declined sharply across successive doses of the multi-dose antigens, with the steepest fall for rotavirus and the later polio and PCV doses (Fig 3).

### 3.10 Model fit and diagnostics

The zero-dose model showed adequate calibration (Hosmer-Lemeshow χ^2^ = 7.66, df = 8, p = 0.47; Table F in S1 Appendix). McFadden’s pseudo-R^2^ values were 0.097 for zero-dose, 0.055 for basic under-immunisation, and 0.025 for national under-immunisation (Table E in S1 Appendix). Likelihood-ratio tests (unweighted approximation) were significant for all three models (zero-dose χ^2^ = 49.5, df = 20, p < 0.001; basic χ^2^ = 120.4, df = 29, p < 0.001; national χ^2^ = 51.7, df = 26, p = 0.002; Table E in S1 Appendix). Influence diagnostics indicated no single observation unduly influenced the zero-dose model estimates (Fig A in S1 Appendix). All GVIF values were below 2.0, indicating no problematic multicollinearity (Table B in S1 Appendix).

### 3.11 Mediation analysis

To explore whether the strong association between household wealth and zero-dose vaccination operates through antenatal care attendance, we conducted a formal mediation analysis. We examined whether poorer women are less likely to reach the WHO-recommended threshold of four or more ANC visits, and whether this in turn reduces vaccination uptake. Using the binary ≥4 ANC visits indicator, we found that ANC attendance explained very little of the wealth effect. The indirect pathway through ANC was not statistically significant (ACME = -0.00087, 95% CI: -0.0025 to 0.0002, p = 0.13), and the estimated proportion of the wealth effect mediated by ANC attendance was near zero (-4.7%, p = 0.20). These findings suggest that the primary mechanism linking poverty to zero-dose status operates through pathways other than antenatal attendance, such as geographic access, opportunity costs, or health system contact at birth. It should be noted that this analysis captures attendance at a minimum threshold (≥4 visits) and cannot assess whether visit timing, content, or quality mediates the wealth-vaccination relationship.

## 4. Discussion

This study identified substantial socioeconomic and geographic inequities in childhood immunization in Zambia. The findings suggest that zero-dose status, incomplete basic immunization, and incomplete national schedule immunization represent distinct patterns of vulnerability with different underlying determinants. Poverty and place of delivery were strongly associated with zero-dose status, while maternal education and vaccination card possession were more strongly associated with completion of the basic immunization schedule.

### Prevalence ratio interpretation

For the common under-immunisation outcomes, prevalence ratios provide a more appropriate measure of association than odds ratios. Re-estimation of associations using survey-weighted Poisson regression demonstrated that the direction and statistical significance of key findings remained unchanged, although the magnitude of associations was attenuated.

For example, maternal secondary education was associated with a prevalence ratio of 0.68 compared with an odds ratio of 0.50, while vaccination card possession yielded a prevalence ratio of 0.53 compared with an odds ratio of 0.24. These findings are consistent with the well-recognised tendency of odds ratios to exaggerate effect sizes when outcomes are common. For public health interpretation, prevalence ratios therefore provide the more informative measure for under-immunisation outcomes.

### Zero-dose children

Among zero-dose children, household wealth is the dominant determinant. The steep wealth gradient reflects the deep structural barriers faced by the poorest families: geographic isolation, the opportunity costs of seeking care, and limited access to information. This gradient aligns with the socioeconomic patterning of immunization access documented across sub-Saharan Africa [12,13]. Facility delivery remained strongly protective even after adjusting for wealth, highlighting the importance of birth-related health system contact as an entry point for vaccination [16,17]. The absence of an independent rural effect once wealth and access were considered suggests that much of the observed rural disadvantage may operate through socioeconomic deprivation and differential access to services. The steep wealth gradient we observed mirrors patterns reported outside sub-Saharan Africa: in South Asian settings such as India and Pakistan, the poorest households and home deliveries similarly drive zero-dose concentration, suggesting the mechanisms are structural rather than region-specific [18,19].

### Basic schedule under-immunisation

Under-immunization within the basic vaccine schedule shows a different pattern. Possession of a vaccination card emerged as the strongest predictor: children whose card was seen had 76% lower adjusted odds of basic under-immunisation (aOR 0.24), corresponding to a 47% lower prevalence (PR 0.53).

Vaccination card availability was strongly associated with basic-schedule completion (PR = 0.53). Similar associations between vaccination-card availability and vaccination coverage or series completion have been reported in other sub-Saharan African settings [20,21]. However, card availability may also reflect previous engagement with immunization services and socioeconomic advantage rather than an independent causal effect of possessing the card itself. Maternal education also remained independently protective [13], indicating that once children enter the system, caregiver knowledge and health-seeking behaviour influence completion of vaccination schedules.

### National schedule under-immunisation

Under-immunization within the expanded national schedule, including PCV and rotavirus vaccines, may reflect supply-side constraints, though the ZDHS did not collect direct measures of vaccine availability. With approximately 70% of children missing at least one of these vaccines and no strong socioeconomic predictors identified, system-level challenges are a plausible explanation. However, without facility-level data on stock-outs, cold chain functionality, or vaccine availability, we cannot confirm this mechanism. Operational research is needed to identify the specific barriers in the Zambian context.

### Antigen-specific patterns

The antigen-level analysis showed substantial differences in schedule completion. Rotavirus dose 2 had the largest coverage gap, with 46.8% of children missing the dose, followed by polio dose 3 (21.7%) and PCV dose 3 (13.4%). Previous research in Zambia documented substantial disruption to rotavirus vaccination during 2021–2023, including Rotarix stock-outs and the subsequent transition to Rotavac [22]. These disruptions provide important context for the low rotavirus coverage observed in the 2024 ZDHS, although the DHS does not contain vaccine-stock or supply-chain data and therefore cannot directly establish the cause of the observed gap. Rotavirus vaccine dropout has also been documented across sub-Saharan Africa and associated with barriers to healthcare access and maternal health-service utilization [23]. The declining coverage observed across successive doses of some multi-dose antigens similarly highlights the importance of strengthening continuity of vaccination services and reducing missed opportunities for vaccination.

### Effect modification by residence

The interaction between wealth and residence highlights the need for differentiated strategies. Urban residence does not guarantee equitable access, and programmes must prioritise disadvantaged urban populations, particularly those in informal settlements. The stronger association between maternal education and vaccine completion observed in rural areas is consistent with previous evidence showing that maternal education is positively associated with childhood vaccination uptake across different geographic settings [24].

### Strengths

This study has several strengths. First, the use of the 2024 ZDHS provides a large, nationally representative descriptive sample (n = 1,738; n = 1,686 in the adjusted models) with high response rates and standardised data collection procedures. Second, the survey-weighted analysis appropriately accounts for the complex survey design, producing nationally generalisable estimates. Third, the disaggregation of immunisation outcomes, zero-dose, basic under-immunisation, and national under-immunisation, reveals distinct patterns with different determinants and programmatic implications. Fourth, we conducted extensive sensitivity analyses, including multiple imputation for missing data, Firth penalised regression for rare outcomes, parsimonious model fitting, and antigen-level coverage analysis. Fifth, the antigen-level analysis provides actionable programmatic data on which specific vaccines are driving the coverage gaps.

### Limitations

Several limitations should be considered. First, the cross-sectional design limits causal inference, although the findings remained consistent across multiple sensitivity analyses. Second, vaccination card possession may reflect not only documentation practices but also broader engagement with the health system, making causal interpretation more complex. Third, the small number of zero-dose children in some provincial subgroups reduced statistical precision, as reflected in the wide confidence intervals for several estimates. Fourth, the survey did not collect direct measures of vaccine supply conditions, such as vaccine stock-outs, cold-chain performance, service readiness, or health worker availability, limiting our ability to directly evaluate supply-side determinants of under-immunisation. Although the high prevalence of missed PCV and rotavirus doses is consistent with possible supply-side constraints, these mechanisms cannot be confirmed using DHS data alone.

Fifth, the zero-dose analysis was based on only 59 events in the complete-case sample, resulting in a low events-per-parameter ratio (EPV = 2.95), below the conventional rule-of-thumb threshold of 10 events per parameter. This raises the possibility of model overfitting and unstable estimates for some covariates. However, several analyses suggest that the principal findings are robust. A parsimonious model produced directionally consistent estimates, Firth penalised logistic regression yielded similar conclusions, and multiple-imputation analyses did not materially alter the associations observed for household wealth and facility delivery. Nevertheless, the low EPV remains an inherent limitation of the available data, and results for sparse subgroups should be interpreted cautiously.

Finally, the survey may have underrepresented highly marginalised populations, including mobile, nomadic, and displaced households, suggesting that the true burden of zero-dose and under-immunised children may be slightly higher than reported.

### Programmatic implications

These findings point to three operationally distinct responses corresponding to the three patterns identified. For zero-dose children, interventions must target the structural determinants of non-contact: poverty, home delivery, and geographic barriers. Outreach strategies, including mobile vaccination teams, community health worker-led household tracing, and demand-generation in the poorest communities, should be intensified within the Reaching Every District framework, with North Western and Northern provinces prioritised.

For basic schedule under-immunization, the strong association with vaccination card possession argues for universal issuance and active retention strategies, including replacement of lost cards at every health contact. Community health worker follow-up for children identified as missing doses would substantially improve completion rates. Investment in maternal literacy and health education programmes would reinforce these gains, particularly in rural settings.

For national schedule under-immunization, demand-side interventions alone are unlikely to be sufficient. The primary response should be supply-side: sustained investment in cold chain infrastructure and maintenance, uninterrupted vaccine stock at facility and outreach level, and strengthened vaccine logistics systems particularly for the later-introduced PCV and rotavirus antigens.

### Recommended actions

Based on these results, we recommend the following priority actions:

1. For zero-dose children: Intensify outreach to the poorest households and those with home deliveries, particularly in North Western and Northern provinces.
2. For basic schedule under-immunisation: Strengthen vaccination card issuance and retention, introduce electronic immunization registries, and expand community health worker follow-up.
3. For national schedule under-immunisation: Address supply-side constraints through cold chain infrastructure investment, real-time stock monitoring, and logistics strengthening.
4. Cross-cutting: Strengthen routine immunization systems as an essential component of pandemic preparedness.

## 5. Conclusions

This study identified three distinct patterns of immunisation gaps in Zambia, each requiring a targeted policy and programmatic response.

For zero-dose children, efforts should focus on reaching the poorest households, children born at home, and provinces with the highest burden, particularly North Western and Northern Provinces. Outreach interventions, including mobile vaccination teams and alternative service delivery approaches, should be strengthened within the Reaching Every District framework.

For children under-immunised on the basic schedule, the priority is improving completion of vaccine doses within the existing routine immunization system. Key interventions include universal issuance and retention of vaccination cards, stronger follow-up through community health workers, and introduction of electronic immunization registries.

For children under-immunised on the national schedule, the largest and most operationally significant gap, supply-side system strengthening is critical. Investments in cold chain infrastructure, real-time stock monitoring, and vaccine logistics are needed to ensure consistent availability of PCV and rotavirus vaccines across all service delivery points.

Two broader priorities cut across all three challenges. First, children born outside health facilities require dedicated tracking and follow-up through community health worker networks. Second, resilient routine immunization systems should be recognised as an essential component of pandemic preparedness.

Without accelerated and equity-focused interventions, Zambia and many comparable low- and middle-income countries are unlikely to achieve Immunization Agenda 2030 and measles elimination targets by 2030. The consistent findings from the 2024 ZDHS provide a clear evidence base and a practical roadmap for strengthening equitable immunization delivery in Zambia.

## Supporting information

S1 Appendix Supplementary tables. Table A. Univariable (survey-weighted Wald) screening of candidate predictors, by outcome. Table B. Generalised variance inflation factors for the three main models. Table C. Multiple-imputation results for the zero-dose model (m = 5). Table D. Odds ratio versus prevalence ratio for the two common outcomes. Table E. Model-fit statistics (unweighted logistic approximations). Table F. Hosmer–Lemeshow goodness-of-fit test (zero-dose model). Table G. Parsimonious zero-dose model (events per variable–respecting). Table H. Causal mediation of the poorest-vs-rest effect on zero-dose status through ≥4 ANC visits. Table I. Antigen missingness among children under-immunised on the national schedule (n = 1,218). Table J. Reconciliation of zero-dose event counts. Table K. Comparison of children included in versus excluded from the complete-case models. Table L. Firth penalised-likelihood zero-dose model (rare-event sensitivity). Supplementary Figures. Fig A. Influence diagnostics for the zero-dose model. DfBeta values by observation for key predictors (facility delivery, any postnatal check, richest wealth, secondary education); dashed red lines mark the ± 2/√n threshold, beyond which observations are potentially influential. Fig B. Distribution of predicted probabilities from the zero-dose model, by observed status. Overlaid density histograms of model-predicted probabilities for children observed as zero-dose versus not zero-dose; separation between the distributions indicates discrimination. Fig C. Stratified forest plot of basic under-immunisation by residence. Adjusted odds ratios (95% CI, log scale) for key predictors estimated separately in urban and rural sub-samples. Urban wealth terms with separation-inflated confidence intervals are omitted from the plot. Fig D. Provincial variation in immunisation outcomes, Zambia DHS 2024. Grouped bar chart of survey-weighted prevalence of zero-dose status and basic and national under-immunisation across the ten provinces, with 95% confidence intervals. Fig E. Sensitivity analysis: complete-case versus multiple imputation, zero-dose model. Adjusted odds ratios (95% CI, log scale) for key zero-dose predictors under complete-case analysis and after multiple imputation (m = 5), showing consistency across approaches.
(DOCX)
