# Supplementary material for: Socioeconomic and health system determinants of zero-dose and under-immunization among children in Zambia: Evidence from the 2024 Demographic and Health Survey
Source: PLOS Glob Public Health. 2026 Oct 30;6(10):e0006618. doi: 10.1371/journal.pgph.0006618 (PMC13722170; doi:10.1371/journal.pgph.0006618)
Supplement: Supplementary file 1 [file pgph.0006618.s001.docx]

**Title**: Socioeconomic and Health System Determinants of Zero-Dose and Under-Immunisation Among Children in Zambia: Evidence from the 2024 Demographic and Health Survey

# S1 Appendix

## Tables

### Table A. Univariable (survey-weighted Wald) screening of candidate predictors, by outcome

| **Variable** | **Wald F** | **p-value** | **Decision** |
| --- | --- | --- | --- |
| **Zero-dose** |  |  |  |
| Household wealth quintile | 4.13 | 0.003 | Entered |
| Maternal education | 3.62 | 0.013 | Entered |
| Residence | 7.46 | 0.007 | Entered |
| Province | 1.50 | 0.144 | Entered |
| Birth order | 1.57 | 0.196 | Entered |
| Preceding birth interval | 1.07 | 0.360 | Not entered |
| Maternal age group | 0.25 | 0.864 | Not entered |
| Child sex | 0.11 | 0.744 | Not entered |
| Facility delivery | 13.60 | <0.001 | Entered |
| Distance a big problem | 0.32 | 0.575 | Not entered |
| ≥ 4 ANC visits | 2.57 | 0.110 | Entered |
| Any postnatal check | 1.88 | 0.171 | Entered |
| Vaccination card seen | 12.00 | <0.001 | Entered |
| ≥ 2 tetanus injections | 0.12 | 0.731 | Not entered |
| Household size | 0.04 | 0.852 | Not entered |
| Religion | — | — | Excluded (separation) |
| Marital status | 0.58 | 0.561 | Not entered |
| Maternal occupation | 3.20 | 0.023 | Entered |
| Financial barrier | 0.02 | 0.904 | Not entered |
| Media exposure | 11.30 | <0.001 | Entered |
| Health insurance | — | — | Excluded (separation) |
| **Under-immunised (basic)** |  |  |  |
| Household wealth quintile | 6.84 | <0.001 | Entered |
| Maternal education | 11.70 | <0.001 | Entered |
| Residence | 12.40 | <0.001 | Entered |
| Province | 1.31 | 0.229 | Not entered |
| Birth order | 2.29 | 0.077 | Entered |
| Preceding birth interval | 2.17 | 0.091 | Entered |
| Maternal age group | 0.10 | 0.960 | Not entered |
| Child sex | 5.26 | 0.022 | Entered |
| Facility delivery | 2.86 | 0.091 | Entered |
| Distance a big problem | 5.38 | 0.021 | Entered |
| ≥ 4 ANC visits | 4.49 | 0.035 | Entered |
| Any postnatal check | 0.94 | 0.334 | Not entered |
| Vaccination card seen | 37.10 | <0.001 | Entered |
| ≥ 2 tetanus injections | 0.46 | 0.498 | Not entered |
| Household size | 0.01 | 0.925 | Not entered |
| Religion | 0.16 | 0.694 | Not entered |
| Marital status | 2.51 | 0.082 | Entered |
| Maternal occupation | 1.42 | 0.237 | Not entered |
| Financial barrier | 0.65 | 0.421 | Not entered |
| Media exposure | 12.50 | <0.001 | Entered |
| Health insurance | 8.04 | 0.005 | Entered |
| **Under-immunised (national)** |  |  |  |
| Household wealth quintile | 2.60 | 0.036 | Entered |
| Maternal education | 4.87 | 0.002 | Entered |
| Residence | 8.24 | 0.004 | Entered |
| Province | 4.28 | <0.001 | Entered |
| Birth order | 1.94 | 0.123 | Entered |
| Preceding birth interval | 0.48 | 0.694 | Not entered |
| Maternal age group | 0.35 | 0.786 | Not entered |
| Child sex | 0.84 | 0.359 | Not entered |
| Facility delivery | 4.24 | 0.040 | Entered |
| Distance a big problem | 2.11 | 0.147 | Entered |
| ≥ 4 ANC visits | 2.80 | 0.095 | Entered |
| Any postnatal check | 0.42 | 0.517 | Not entered |
| Vaccination card seen | 3.54 | 0.061 | Entered |
| ≥ 2 tetanus injections | 0.91 | 0.340 | Not entered |
| Household size | 3.30 | 0.070 | Entered |
| Religion | 2.86 | 0.091 | Entered |
| Marital status | 1.98 | 0.139 | Entered |
| Maternal occupation | 0.98 | 0.400 | Not entered |
| Financial barrier | 2.10 | 0.148 | Entered |
| Media exposure | 2.46 | 0.117 | Entered |
| Health insurance | 3.97 | 0.047 | Entered |

***Footnote:*** *Univariable survey-weighted associations (Wald F test). Variables with p < 0.20 were considered for the multivariable models alongside a-priori confounders (household wealth, residence). Religion and health insurance were excluded a priori from the zero-dose model because of quasi-complete separation (no zero-dose events in one category); univariable statistics are therefore not reported for those cells.*

### Table B. Generalised variance inflation factors for the three main models

| **Variable** | **GVIF^(1/2·df)** |
| --- | --- |
| **Zero-dose model** |  |
| Household wealth quintile | 1.61 |
| Maternal education | 1.62 |
| Residence | 1.66 |
| Birth order | 1.66 |
| Maternal age group | 1.46 |
| Child sex | 1.49 |
| Facility delivery | 1.25 |
| Distance a big problem | 1.25 |
| ≥ 4 ANC visits | 1.22 |
| Any postnatal check | 1.57 |
| Household size | 1.22 |
| **Basic under-immunisation model** |  |
| Household wealth quintile | 1.21 |
| Maternal education | 1.15 |
| Residence | 1.62 |
| Religion | 1.03 |
| Marital status | 1.17 |
| Maternal occupation | 1.11 |
| Birth order | 1.35 |
| Maternal age group | 1.33 |
| Child sex | 1.11 |
| Facility delivery | 1.09 |
| Distance a big problem | 1.11 |
| ≥ 4 ANC visits | 1.06 |
| Any postnatal check | 1.10 |
| Household size | 1.22 |
| Financial barrier | 1.06 |
| Vaccination card seen | 1.06 |
| ≥ 2 tetanus injections | 1.06 |
| **National under-immunisation model** |  |
| Household wealth quintile | 1.22 |
| Maternal education | 1.15 |
| Residence | 1.57 |
| Religion | 1.03 |
| Marital status | 1.20 |
| Maternal occupation | 1.12 |
| Birth order | 1.33 |
| Maternal age group | 1.31 |
| Child sex | 1.06 |
| Facility delivery | 1.09 |
| Distance a big problem | 1.08 |
| ≥ 4 ANC visits | 1.06 |
| Any postnatal check | 1.10 |
| Household size | 1.22 |
| Financial barrier | 1.06 |
| Vaccination card seen | 1.06 |
| ≥ 2 tetanus injections | 1.03 |

***Footnote:*** *Generalised variance inflation factors, GVIF^(1/(2·df)). All values are below 2.0, indicating no problematic multicollinearity in any model.*

**Table C. Multiple-imputation results for the zero-dose model (m = 5)**

| **Term** | **aOR** | **95% CI** | **p** |
| --- | --- | --- | --- |
| Intercept | 0.36 | 0.07–1.84 | 0.220 |
| Wealth: Poorer | 0.52 | 0.25–1.07 | 0.078 |
| Wealth: Middle | 0.75 | 0.35–1.60 | 0.458 |
| Wealth: Richer | 0.20 | 0.05–0.86 | 0.031 |
| Wealth: Richest | 0.08 | 0.01–1.05 | 0.055 |
| Education: Primary | 1.32 | 0.53–3.27 | 0.554 |
| Education: Secondary | 0.63 | 0.21–1.91 | 0.415 |
| Education: Higher | 1.91 | 0.14–26.84 | 0.633 |
| Rural (vs urban) | 0.91 | 0.38–2.16 | 0.822 |
| Birth order 2–3 | 0.58 | 0.26–1.29 | 0.181 |
| Birth order 4–5 | 0.58 | 0.20–1.69 | 0.320 |
| Birth order 6+ | 0.95 | 0.25–3.65 | 0.935 |
| Maternal age 20–29 | 1.11 | 0.46–2.65 | 0.822 |
| Maternal age 30–39 | 1.07 | 0.30–3.84 | 0.922 |
| Maternal age 40+ | 0.85 | 0.17–4.36 | 0.844 |
| Facility delivery | 0.29 | 0.14–0.59 | <0.001 |
| Distance a big problem | 0.73 | 0.41–1.29 | 0.275 |
| ≥ 4 ANC visits | 0.63 | 0.33–1.20 | 0.157 |
| Any postnatal check | 0.60 | 0.32–1.15 | 0.124 |
| Household size | 1.03 | 0.93–1.14 | 0.552 |

***Footnote:*** *Pooled estimates from m = 5 multiply-imputed datasets (predictive mean matching; Rubin's rules; seed 20260112). Results are consistent with the complete-case model in Table 3.*

**Table D. Odds ratio versus prevalence ratio for the two common outcomes**

| **Term** | **OR** | **OR 95% CI** | **PR** | **PR 95% CI** |
| --- | --- | --- | --- | --- |
| **Under-immunised (basic)** |  |  |  |  |
| Wealth: Poorer | 0.95 | 0.68–1.33 | 0.97 | 0.81–1.17 |
| Wealth: Middle | 0.90 | 0.63–1.28 | 0.94 | 0.77–1.15 |
| Wealth: Richer | 0.59 | 0.36–0.98 | 0.71 | 0.52–0.99 |
| Wealth: Richest | 0.56 | 0.31–1.01 | 0.69 | 0.47–1.01 |
| Education: Primary | 0.70 | 0.45–1.09 | 0.84 | 0.68–1.04 |
| Education: Secondary | 0.50 | 0.30–0.82 | 0.68 | 0.52–0.88 |
| Education: Higher | 0.46 | 0.22–0.95 | 0.64 | 0.40–1.03 |
| Rural (vs urban) | 0.91 | 0.64–1.31 | 0.94 | 0.75–1.17 |
| Non-Christian | 1.14 | 0.29–4.47 | 1.08 | 0.50–2.32 |
| Married/cohabiting | 1.13 | 0.76–1.69 | 1.09 | 0.85–1.39 |
| Previously married | 1.40 | 0.87–2.26 | 1.23 | 0.92–1.64 |
| White collar / services | 1.00 | 0.74–1.34 | 1.00 | 0.83–1.20 |
| Agricultural | 0.81 | 0.62–1.07 | 0.88 | 0.75–1.04 |
| Manual labour | 0.79 | 0.48–1.29 | 0.87 | 0.65–1.17 |
| Birth order 2–3 | 1.00 | 0.72–1.39 | 1.00 | 0.82–1.23 |
| Birth order 4–5 | 0.98 | 0.63–1.54 | 0.99 | 0.75–1.30 |
| Birth order 6+ | 1.17 | 0.64–2.12 | 1.08 | 0.76–1.53 |
| Maternal age 20–29 | 1.07 | 0.70–1.64 | 1.04 | 0.80–1.35 |
| Maternal age 30–39 | 1.08 | 0.63–1.85 | 1.04 | 0.75–1.45 |
| Maternal age 40+ | 0.95 | 0.47–1.90 | 0.97 | 0.64–1.46 |
| Female (vs male) | 1.28 | 1.03–1.60 | 1.16 | 1.02–1.33 |
| Facility delivery | 0.95 | 0.60–1.52 | 0.99 | 0.78–1.26 |
| Distance a big problem | 1.01 | 0.80–1.28 | 1.01 | 0.88–1.15 |
| ≥ 4 ANC visits | 0.78 | 0.58–1.05 | 0.87 | 0.73–1.03 |
| Any postnatal check | 0.94 | 0.72–1.23 | 0.96 | 0.82–1.13 |
| Household size | 1.00 | 0.95–1.05 | 1.00 | 0.97–1.03 |
| Financial barrier | 0.97 | 0.53–1.77 | 0.98 | 0.71–1.36 |
| Vaccination card seen | 0.24 | 0.14–0.40 | 0.53 | 0.44–0.63 |
| ≥ 2 tetanus injections | 0.98 | 0.78–1.24 | 0.99 | 0.86–1.14 |
| **Under-immunised (national)** |  |  |  |  |
| Wealth: Poorer | 1.08 | 0.76–1.55 | 1.02 | 0.93–1.12 |
| Wealth: Middle | 0.92 | 0.63–1.37 | 0.98 | 0.88–1.09 |
| Wealth: Richer | 0.86 | 0.52–1.44 | 0.96 | 0.82–1.12 |
| Wealth: Richest | 0.85 | 0.45–1.57 | 0.95 | 0.78–1.15 |
| Education: Primary | 0.86 | 0.52–1.42 | 0.96 | 0.86–1.08 |
| Education: Secondary | 0.73 | 0.42–1.29 | 0.92 | 0.80–1.06 |
| Education: Higher | 0.44 | 0.21–0.93 | 0.76 | 0.59–0.98 |
| Rural (vs urban) | 1.17 | 0.80–1.72 | 1.05 | 0.94–1.18 |
| Non-Christian | 0.32 | 0.09–1.14 | 0.61 | 0.29–1.29 |
| Married/cohabiting | 1.25 | 0.83–1.87 | 1.07 | 0.94–1.22 |
| Previously married | 1.01 | 0.60–1.69 | 1.00 | 0.85–1.18 |
| White collar / services | 1.25 | 0.91–1.70 | 1.07 | 0.97–1.18 |
| Agricultural | 1.08 | 0.77–1.53 | 1.02 | 0.93–1.12 |
| Manual labour | 1.10 | 0.64–1.89 | 1.03 | 0.88–1.20 |
| Birth order 2–3 | 0.73 | 0.51–1.05 | 0.91 | 0.82–1.01 |
| Birth order 4–5 | 0.88 | 0.56–1.39 | 0.97 | 0.85–1.10 |
| Birth order 6+ | 0.99 | 0.52–1.88 | 1.00 | 0.83–1.19 |
| Maternal age 20–29 | 1.22 | 0.80–1.86 | 1.06 | 0.93–1.21 |
| Maternal age 30–39 | 1.27 | 0.71–2.29 | 1.07 | 0.90–1.29 |
| Maternal age 40+ | 1.28 | 0.60–2.70 | 1.08 | 0.87–1.34 |
| Female (vs male) | 1.13 | 0.89–1.43 | 1.04 | 0.97–1.11 |
| Facility delivery | 0.79 | 0.50–1.26 | 0.95 | 0.86–1.05 |
| Distance a big problem | 0.96 | 0.75–1.24 | 0.99 | 0.92–1.06 |
| ≥ 4 ANC visits | 0.77 | 0.55–1.08 | 0.93 | 0.86–1.02 |
| Any postnatal check | 1.09 | 0.83–1.44 | 1.03 | 0.95–1.11 |
| Household size | 0.96 | 0.91–1.00 | 0.99 | 0.97–1.00 |
| Financial barrier | 1.42 | 0.76–2.64 | 1.09 | 0.95–1.24 |
| Vaccination card seen | 0.65 | 0.37–1.13 | 0.90 | 0.80–1.01 |
| ≥ 2 tetanus injections | 0.91 | 0.70–1.19 | 0.97 | 0.90–1.06 |

***Footnote:*** *Odds ratios (OR) from survey-weighted logistic regression and prevalence ratios (PR) from survey-weighted Poisson regression with robust standard errors, using the same covariate set. PRs are the primary effect measure for these common outcomes and are attenuated relative to ORs, as expected.*

**Table E. Model-fit statistics (unweighted logistic approximations)**

| **Outcome** | **McFadden R²** | **Nagelkerke R²** | **LR χ²** | **df** | **LR p** | **AIC** |
| --- | --- | --- | --- | --- | --- | --- |
| Zero-dose | 0.097 | 0.111 | 49.5 | 20 | < 0.001 | 504 |
| Under-immunised (basic) | 0.055 | 0.094 | 120.4 | 29 | < 0.001 | 2,141 |
| Under-immunised (national) | 0.028 | 0.048 | 57.7 | 29 | < 0.001 | 2,069 |

***Footnote:*** *Pseudo-R², likelihood-ratio (LR) tests and AIC computed from the corresponding unweighted logistic models, reported as approximations because svyglm has no valid likelihood. LR = likelihood-ratio test against the null model.*

**Table F. Hosmer–Lemeshow goodness-of-fit test (zero-dose model)**

| **Model** | **χ²** | **df** | **p-value** |
| --- | --- | --- | --- |
| Zero-dose | 7.66 | 8 | 0.467 |

***Footnote:*** *Hosmer–Lemeshow test with 10 groups, applied to unweighted fitted values as an approximation; p > 0.05 indicates no evidence of poor fit.*

**Table G. Parsimonious zero-dose model (events per variable–respecting)**

| **Term** | **aOR** | **95% CI** | **p** |
| --- | --- | --- | --- |
| Wealth: Poorer | 0.51 | 0.26–1.00 | 0.051 |
| Wealth: Middle | 0.69 | 0.32–1.48 | 0.337 |
| Wealth: Richer | 0.26 | 0.06–1.02 | 0.054 |
| Wealth: Richest | 0.08 | 0.02–0.33 | <0.001 |
| Rural (vs urban) | 0.90 | 0.38–2.12 | 0.815 |
| Education: Primary | 1.32 | 0.55–3.21 | 0.536 |
| Education: Secondary | 0.79 | 0.26–2.37 | 0.674 |
| Education: Higher | 2.68 | 0.45–16.01 | 0.278 |
| Birth order 2–3 | 0.63 | 0.31–1.29 | 0.209 |
| Birth order 4–5 | 0.65 | 0.30–1.45 | 0.295 |
| Birth order 6+ | 1.00 | 0.41–2.41 | 0.991 |
| Facility delivery | 0.36 | 0.17–0.75 | 0.007 |
| ≥ 4 ANC visits | 0.71 | 0.38–1.32 | 0.278 |
| Any postnatal check | 0.58 | 0.27–1.23 | 0.154 |
| Vaccination card seen | 0.37 | 0.17–0.78 | 0.010 |

***Footnote:*** *Survey-weighted parsimonious zero-dose model retaining univariable-screened predictors plus a-priori confounders (household wealth, residence). Events per variable improved from 2.95 to 3.93. The wealth gradient and facility-delivery association are preserved.*

**Table H. Causal mediation of the poorest-vs-rest effect on zero-dose status through ≥4 ANC visits**

| **Quantity** | **Estimate** | **95% CI** | **p** |
| --- | --- | --- | --- |
| ACME (average) | −0.00086 | −0.0026 to 0.0002 | 0.170 |
| ADE (average) | 0.0191 | −0.0020 to 0.0456 | 0.080 |
| Total effect | 0.0182 | −0.0026 to 0.0442 | 0.098 |
| Proportion mediated (average) | −0.047 | −0.397 to 0.208 | 0.252 |

***Footnote:*** *Causal mediation analysis (Imai et al.; nonparametric bootstrap, 1,000 resamples; seed 20260112). ACME = average causal mediation effect; ADE = average direct effect. The indirect pathway through ANC attendance is small and non-significant.*

**Table I. Antigen missingness among children under-immunised on the national schedule (n = 1,218)**

| **Vaccine (dose)** | **n missing** | **% of under-immunised** |
| --- | --- | --- |
| Rotavirus 2 | 824 | 67.7 |
| Rotavirus 1 | 567 | 46.6 |
| OPV 3 | 370 | 30.4 |
| PCV 3 | 233 | 19.1 |
| Measles (MCV1) | 218 | 17.9 |
| OPV 2 | 166 | 13.6 |
| PCV 2 | 136 | 11.2 |
| OPV 1 | 118 | 9.7 |
| BCG | 97 | 8.0 |
| PCV 1 | 95 | 7.8 |
| Pentavalent 3 | 93 | 7.6 |
| Pentavalent 1 | 62 | 5.1 |
| Pentavalent 2 | 60 | 4.9 |

***Footnote:*** *Among the 1,218 children under-immunised on the national schedule, the percentage lacking each antigen (unweighted counts). Rotavirus and later multi-dose antigens dominate the coverage gap.*

**Table J. Reconciliation of zero-dose event counts**

| **Sample** | **n** | **Zero-dose events** | **Used for** |
| --- | --- | --- | --- |
| Full descriptive sample | 1,738 | 62 | Table 1; prevalence |
| Complete-case modelling sample | 1,686 | 59 | Regression / EPV / Firth / MI |
| Difference | 52 | 3 | Dropped (≥1 missing covariate) |

***Footnote:*** *Both counts are correct for their respective denominators: 62 zero-dose children in the full descriptive sample (N = 1,738) and 59 in the complete-case modelling sample (N = 1,686); three zero-dose children were dropped for missing covariates. EPV = events per variable; MI = multiple imputation.*

**Table K. Comparison of children included in versus excluded from the complete-case models**

| **Characteristic** | **Excluded N = 34** | **Included N = 1,704** | **p-value** |
| --- | --- | --- | --- |
| **Household wealth quintile** |  |  | **0.4** |
| Poorest | 13 (38%) | 462 (27%) |  |
| Poorer | 9 (26%) | 397 (23%) |  |
| Middle | 3 (8.8%) | 345 (20%) |  |
| Richer | 6 (18%) | 286 (17%) |  |
| Richest | 3 (8.8%) | 214 (13%) |  |
| **Maternal education** |  |  | **0.2** |
| No education | 1 (2.9%) | 133 (7.8%) |  |
| Primary | 21 (62%) | 805 (47%) |  |
| Secondary | 9 (26%) | 675 (40%) |  |
| Higher | 3 (8.8%) | 91 (5.3%) |  |
| **Residence** |  |  | **0.14** |
| Urban | 7 (21%) | 574 (34%) |  |
| Rural | 27 (79%) | 1,130 (66%) |  |
| Zero-dose | 3 (8.8%) | 59 (3.5%) | 0.12 |
| Under-immunised (basic) | 16 (47%) | 610 (36%) | 0.2 |
| Under-immunised (national) | 27 (79%) | 1,191 (70%) | 0.3 |

***Footnote:*** *p-values from Fisher's exact tests (Monte-Carlo simulation for categorical variables). No characteristic differed at p < 0.05, supporting the missing-at-random assumption. Note: this comparison uses a child-ID (cluster–household–birth) match that classifies 34 excluded / 1,704 included; the definitive analytic exclusion by any missing covariate is 52 / 1,686 (Table J).*

**Table L. Firth penalised-likelihood zero-dose model (rare-event sensitivity)**

| **Term** | **aOR** | **95% CI** | **p** |
| --- | --- | --- | --- |
| Wealth: Poorer | 0.54 | 0.26–1.08 | 0.081 |
| Wealth: Middle | 0.78 | 0.36–1.60 | 0.505 |
| Wealth: Richer | 0.24 | 0.05–0.86 | 0.027 |
| Wealth: Richest | 0.11 | 0.01–0.86 | 0.033 |
| Education: Primary | 1.23 | 0.55–3.23 | 0.632 |
| Education: Secondary | 0.62 | 0.22–1.88 | 0.382 |
| Education: Higher | 2.28 | 0.15–20.55 | 0.523 |
| Rural (vs urban) | 0.90 | 0.39–2.19 | 0.804 |
| Maternal age 20–29 | 1.09 | 0.47–2.62 | 0.841 |
| Maternal age 30–39 | 1.07 | 0.30–3.74 | 0.915 |
| Maternal age 40+ | 0.90 | 0.18–4.28 | 0.897 |
| Birth order 2–3 | 0.59 | 0.27–1.29 | 0.184 |
| Birth order 4–5 | 0.60 | 0.21–1.66 | 0.326 |
| Birth order 6+ | 0.93 | 0.25–3.55 | 0.919 |
| Female (vs male) | 0.92 | 0.55–1.56 | 0.768 |
| Facility delivery | 0.29 | 0.15–0.60 | 0.001 |
| Distance a big problem | 0.74 | 0.41–1.28 | 0.283 |
| ≥ 4 ANC visits | 0.62 | 0.34–1.21 | 0.155 |
| Any postnatal check | 0.62 | 0.32–1.13 | 0.124 |
| Household size | 1.04 | 0.93–1.14 | 0.503 |

***Footnote:*** *Firth penalised-likelihood logistic regression for the zero-dose model (sensitivity for rare events and separation). The wealth gradient and facility-delivery association are preserved, confirming the main-model findings.*

**Supplementary figures**

**Fig A.** Influence diagnostics for the zero-dose model. DfBeta values by observation for key predictors (facility delivery, any postnatal check, richest wealth, secondary education); dashed red lines mark the ±2/√n threshold, beyond which observations are potentially influential.

**Fig B.** Distribution of predicted probabilities from the zero-dose model, by observed status. Overlaid density histograms of model-predicted probabilities for children observed as zero-dose versus not zero-dose; separation between the distributions indicates discrimination.

**Fig C.** Stratified forest plot of basic under-immunisation by residence. Adjusted odds ratios (95% CI, log scale) for key predictors estimated separately in urban and rural sub-samples. Urban wealth terms with separation-inflated confidence intervals are omitted from the plot.

**Fig D.** Provincial variation in immunisation outcomes, Zambia DHS 2024. Grouped bar chart of survey-weighted prevalence of zero-dose status and basic and national under-immunisation across the ten provinces, with 95% confidence intervals.

**Fig E.** Sensitivity analysis: complete-case versus multiple imputation, zero-dose model. Adjusted odds ratios (95% CI, log scale) for key zero-dose predictors under complete-case analysis and after multiple imputation (m = 5), showing consistency across approaches.
